# Roadmap for new practitioners to navigate the multiple myeloma landscape

**DOI:** 10.1016/j.heliyon.2022.e10586

**Published:** 2022-09-12

**Authors:** Tiffany Tam, Eric Smith, Evelyn Lozoya, Hayley Heers, P. Andrew Allred

**Affiliations:** Banner MD Anderson Cancer Center, 2946 E Banner Gateway Drive, Gilbert, AZ 85295, United States

**Keywords:** Multiple myeloma, Immunomodulators, Proteosome inhibitors, Anti-CD38-targeting antibodies

## Abstract

Multiple myeloma (MM) is a blood cancer in which monoclonal plasma cells cause end organ damage resulting in hypercalcemia, renal failure, anemia, and bone lesions. MM is considered incurable, however, recent advances in treatment have improved survival. Historically, MM has been treated with immunomodulatory drugs (IMiDs), proteosome inhibitors (PIs), and corticosteroids. While newer therapeutic approaches such as monoclonal antibodies and cellular therapies have broadened the treatment horizon, the selection and sequencing of these therapies has become more complex. This review aims to help advanced practice providers navigate through the diagnosis, staging, treatment, and supportive care considerations in the MM space.

## 1 Pathophysiology and diagnosis

Multiple myeloma (MM) is a malignancy that accounts for 1% of all malignancies and 10% of all hematologic malignancies [1]. The pathophysiology of MM is described most simply by the overproduction of aberrant plasma cells that produce monoclonal complete or partial light chain immunoglobulins [1]. These dysfunctional plasma cells arise from one or more mutations of which most commonly include deletion (1p), amplification (1q), deletion (13q), deletion (14q), deletion (16q), and mutations within the *RAS, DIS3, FGFR3*, and *TP53* genes [2]. Characterized by monoclonal plasma cell proliferation within the bone marrow, MM has defining features of hypercalcemia, renal insufficiency, anemia, and bony lesions in the skeleton or soft tissue. Of note, bony lesions are attributed to upregulated osteoclast activity and inhibition of osteoblast activity, an event that also contributes to hypercalcemia and renal insufficiency. Renal insufficiency also occurs due to immunoglobulin-mediated cast nephropathy. The cause of anemia in this setting is multifold, caused by both disrupted erythropoiesis secondary to crowding of the bone marrow with dysfunctional plasma cells and due to renal injury. Recently additional defining features have been identified which include myeloma including 60% or more plasmacytosis in the bone marrow, light chain ratio of >100 or <0.01, and more than one myelomatous lesion identified by MRI of emergency [3]. These criteria can be remembered with the “SLiM-CRAB”, acronym and the presence of any necessitate initiation of treatment to reverse end organ damage as defined by the following: (1) Sixty (60%) plasmacytosis, (2) Light chain ratio >100, (3) MRI showing one or more focal lesion, (4) hypercalcemia, (5) renal insufficiency, (6) anemia, and (7) bone disease.

**Figure 1:**
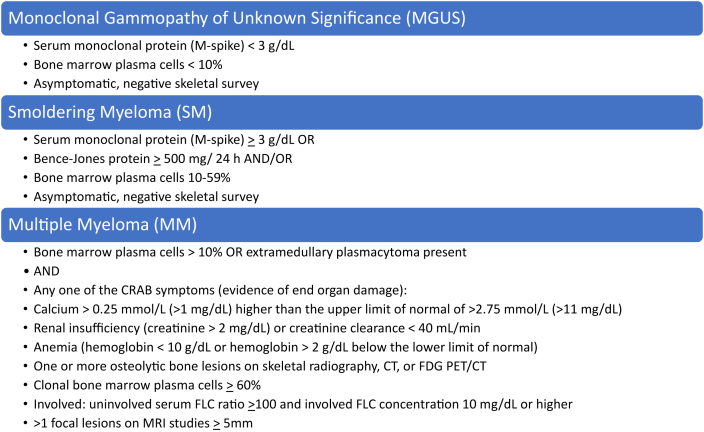
Definitions of monoclonal gammopathy of unknown significance, smoldering myeloma, and multiple myeloma.

MM is thought to arise from premalignant conditions known as monoclonal gammopathy of undetermined significance (MGUS) or smoldering multiple myeloma (SMM) [4]. Each of these conditions are considered asymptomatic as there is no evidence of end organ damage. MGUS has a cumulative 1% risk per year of progressing to MM and SMM has a higher risk of 10 % per year for the first 5 years [5]. MGUS is defined as presence of serum monoclonal protein with monoclonal spike < 3 g/dL and <10% clonal plasmacytosis in the bone marrow [2]. SMM is defined by the presence of serum monoclonal protein with monoclonal spike >3 g/dL and between 10% and 59% clonal plasmacytosis in the bone marrow (see Figure 1) [2].

## 2 Staging and prognosis

Without treatment, the median survival for a patient with newly diagnosed MM is 12 months. With treatment, prognosis is based on a risk stratification approach. The Revised International Staging System (R-ISS) (see Table 1) is the latest evidence-based risk stratification system for MM. In comparison to previous MM staging systems which exclusively relied on biomarkers such as serum albumin, beta-2 microglobulin, and serum lactate dehydrogenase, this system also takes into consideration high-risk cytogenetic abnormalities such as deletion 17p, translocations of chromosome 14, and gains of 1q [6]. To put into perspective, a patient with R-ISS stage III MM would be characterized by having elevated beta-2-microglobulin of 5.5 mg/dL or higher, the presence of high-risk cytogenetics, and/or elevated lactate dehydrogenase. Assuming standard of care treatment is properly administered, median survival ranges from 29 months for high-risk patients to 66 months for standard-risk patients.

**Table 1:** Staging.

Stage	International Staging System (ISS)	Revised-ISS (R-ISS)
I	Serum B2M < 3.5 mg/L Serum albumin ≥3.5 g/dL	ISS stage I (left) **AND** standard-risk cytogenetics by FISH **AND** normal serum LDH
II	Not ISS stage I or III	Not R-ISS stage I or III
III	Serum B2M ≥ 5.5 mg/dL	ISS stage III (left) AND high-risk cytogenetics by FISH **OR** Elevated serum LDH

*Abbreviations:* B2M: beta-2-microglobulin, LDH: lactate dehydrogenase, FISH: fluorescence in situ hybridization.

*Notes:* High risk cytogenetics includes one of the following: deletion (17p), translocation (4; 14), translocation (14; 16).

## 3 Approach to treatment

The backbone of first-line MM therapy consists of a corticosteroid (typically dexamethasone), an immunomodulators (IMiD), and a proteosome inhibitor (PI). Table 2 summarizes key information for the pharmacologic agents used in treating MM. Tables 3 and 4 outline the NCCN-recommended first-line and subsequent-line regimens. Considerations prior to starting therapy include risk stratification, cytogenetics, toxicity profile, feasibility, and affordability.

**Table 2:** Multiple myeloma therapy overview.

Class	Drug	Dosing/Administration	Renal/Hepatic Considerations	Adverse Reactions	Clinical Pearls	Hypersensitivity Reactions
**IMiDs** [7, 8, 9]	**Thalidomide (Thalomid)**	• 50–200 mg by mouth daily continuously in evening at least 1 h after evening meal (200 mg usual starting dose)	• No specific recommendations	• Birth defects/fetal death • VTE • Peripheral neuropathy • Constipation • Sedation	• VTE prophylaxis required • REMS program present • Taken continuously without breaks	Not applicable
	**Lenalidomide (Revlimid or Generic Equivalent)**	• 2.5–25 mg by mouth with or without food days 1–21 every 28d (25 mg usual starting dose)	• Renal: dose adjustments vary depending on indication for CrCl <60 mL/min	• Birth defects/fetal death • VTE • Cytopenias • Peripheral neuropathy • Pruritus, skin rash • Diarrhea, constipation • Muscle spasms	• VTE prophylaxis required • REMS program present • 7-day treatment free period in induction regimens to allow for WBC count recovery	Not applicable
	**Pomalidomide (Pomalyst)**	• 1–4 mg by mouth with or without food days 1–21 every 28d (4 mg usual starting dose)	• Renal: reduce dose to 3 mg with hemodialysis patients or if CrCl <15 mL/min • Hepatic: reduce dose to 3 mg for Child-Pugh A or B; reduce dose to 2 mg for Child-Pugh C	• Birth defects/fetal death • VTE • Cytopenias • Peripheral neuropathy • Pruritus, skin rash • Diarrhea, constipation	• VTE prophylaxis required • REMS program present • 7-day treatment free period in induction regimens to allow for WBC count recovery	Not applicable
**PIs** [10, 11, 12]	**Bortezomib (Velcade)**	• 1.3 mg/m^2^ SQ d1, 4, 8, 11 every 21d OR 1.3 mg/m^2^ d1, 8, 15, 22 every 28d	• Hepatic: reduce starting dose to 0.7 mg/m^2^ for total bilirubin >1.5x ULN; may consider dose escalation to 1 mg/m^2^ in subsequent cycles depending on tolerability	• Thrombocytopenia • Hepatotoxicity • Peripheral neuropathy • Diarrhea, constipation	• Consecutive doses should be separated by at least 72 h • Less peripheral neuropathy with SQ route of administration • HSV/VZV viral prophylaxis required	Not applicable
	**Carfilzomib (Kyprolis)**	• Once-weekly scheme: (cycle 1) 20 mg/m^2^ IV d1, 70 mg/m^2^ d8, 15; (subsequent cycles) 70 mg/m^2^ d1, 8, 15 every 28d • Twice-weekly scheme: (cycle 1) 20 mg/m^2^ IV d1, 2 then 36 mg/m^2^ d8, 9, 15, 16 every 28d; (subsequent cycles) 36 mg/m^2^ d1, 2, 8, 9, 15, 16 every 28d	• Hepatic: reduce dose by 25% for total bilirubin of > 1–3x ULN with any AST or for any AST > ULN; no recommendations provided for total bilirubin of >3 x ULN	• TLS • Cardiotoxicity (heart failure) • Pulmonary complications • Peripheral neuropathy • Cytopenias	• Hydration with 250–500 mL of IV fluid recommended prior to each dose of cycle 1 due to TLS risk; allopurinol not routinely recommended • Administer dexamethasone 30 min to 4 h prior to each carfilzomib dose; counsel patients to take their treatment dexamethasone dose prior to their infusion appointment • Baseline echocardiogram not required but recommended • HSV/VZV viral prophylaxis required	• Reinstate dexamethasone premedication. • No specific recommendations on infusion rate restart
	**Ixazomib (Ninlaro)**	• 4 mg PO d1, 8, 15 every 28d	• Hepatic: reduce starting dose to 3 mg in patients with total bilirubin >1.5–3x • Renal: reduce starting dose to 3 mg in CrCl <30 mL/min or ESRD	• GI toxicities • Peripheral neuropathy • Peripheral edema • Cutaneous reactions • Hepatotoxicity • Cytopenias	• Take on an empty stomach • HSV/VZV viral prophylaxis required	• Not applicable
**Monoclonal Antibodies** [13, 14,15, 16, 17]	**Elotuzumab (Empliciti)**	With lenalidomide: • C1-2: 10 mg/kg IV d1, 8, 15, 22 • C2 onwards: 10 mg/kg IV d1, 15 With pomalidomide: • C1-2: 10 mg/kg IV d1, 8, 15, 22 • C2 onwards: 20 mg/kg IV d1	• Hepatic (transaminitis grade 3 or higher): withhold therapy until resolution	• Hypersensitivity reactions • Infections • Second primary malignancies • Hepatotoxicity • Interference with M-protein	• Premedicate with dexamethasone, acetaminophen, H1RA, and H2RA; 8–28 mg of total weekly dexamethasone dose should be given 3–24 h prior to elotuzumab depending on target weekly dexamethasone dose (see package insert for full details) • Infusion rate titrated based on tolerability	• Upon resolution to Grade 1, restart at 0.5 mL/min and gradually increase at a rate of 0.5 mL/min every 30 min as tolerated to the rate at which the hypersensitivity reaction occurred. Resume escalation if there is no recurrence of reaction.
	**Daratumumab (Darzalex[IV] or Darzalex Faspro [SQ])**	• C1: 8 mg/kg IV d1, 2, 16 mg/kg IV d8, 15, 22 • C2: 16 mg/kg IV d1, 8, 15, 22 • C3-6: 16 mg/kg IV d1, 15 • C7 onwards: 16 mg/kg IV monthly	• No specific recommendations	• Hypersensitivity reactions • Hypertension • Upper and lower respiratory tract infections, cough, bronchitis	• HSV/VZV viral prophylaxis required • Premedicate with dexamethasone, acetaminophen, and H1RA. Montelukast optional. • Infusion rate titrated based on tolerability • May cause false positive reactions in indirect antiglobulin tests (Coombs' test); obtain RBC type and screen prior to first dose	• For daratumumab: • Once reaction symptoms resolve, consider restarting the infusion at no more than half the rate at which the reaction occurred. • Future cycles initiated at 50 mL/h. • For daratumumab and hyaluronidase-fihj: • Pause or slow down delivery rate if the patient experiences pain. In the event pain is not alleviated by pausing or slowing down delivery rate, a second injection site may be chosen on the opposite side of the abdomen to deliver the remainder of the dose.
	**Isatuximab-irfc (Sarclisa)**	• C1: 10 mg/kg IV d1, 8, 15, 22 • C2 onwards: 10 mg/kg IV d1, 15	• No specific recommendations	• Hypersensitivity reactions • Hypertenstion • Upper and lower respiratory infections, dyspnea • Neutropenia	• HSV/VZV viral prophylaxis required • Premedicate with dexamethasone, acetaminophen, H1RA, and H2RA • Infusion rate titrated based on tolerability • May cause false positive reactions in indirect antiglobulin tests (Coombs' test); obtain RBC type and screen prior to first dose	• If symptoms improve, restart at half the initial rate, with supportive care and close monitoring. • If symptoms do not recur after 30 min, the rate may be increased to the initial rate, and then increased incrementally.
	**Belantamab mafodotin-blmf (Blenrep)**	• 2.5 mg/kg IV every 21d	• No specific recommendations	• Ocular toxicity- blurred vision, keratitis, photophobia • Fevers • Thrombocytopenia	• REMS program: routine eye exams required (baseline, prior to each dose, and for worsening ocular symptoms) • Use of preservative-free lubricating eye drops four times daily during therapy recommended • Avoid contact lenses if possible	• If grade 2 or worse, stop the infusion and provide supportive care. Once symptoms resolve, resume at lower infusion rate, at least reduced by 50%.
**Miscellaneous** [18, 19]	**Selinexor (Xpovio)**	With dexamethasone: • 80 mg by mouth d1-3 every 7d (with or without food) With bortezomib and dexamethasone: • 100 mg by mouth every 7d	• No specific recommendations	• Thrombocytopenia • Neutropenia • GI toxicity • Hyponatremia • Neurological toxicity	• Associated with moderate or high emetic potential; antiemetics are recommended to prevent nausea and vomiting (Administer a 5-HT3 antagonist and other antiemetics as clinically appropriate)	• Not applicable
	**Venetoclax (Venclexta)**	• 800 mg once daily	• Hepatic: reduce the daily venetoclax dose by 50% for severe impairment (Child-Pugh class C) • Renal: no specific recommendations	• Edema • Skin rash • Electrolyte disorder • Anemia • Leukopenia • Neutropenia • Thrombocytopenia • Hepatotoxicity • Upper respiratory tract infection	• Associated with many drug-drug interactions including CYP3A4 inducers & inhibitors and P-glycoprotein inhibitors (may constitute dose reductions)	• Not applicable

*Abbreviations:* CrCl: creatinine clearance, ESRD: end-stage renal disease, GI: gastrointestinal, H1RA: histamine type 1 receptor antagonist; H2RA: histamine type 2 receptor antagonist, HSV/VZV: herpes simplex virus/varicella zoster virus, IMiDs: immunomodulators, PIs: proteosome inhibitors, LFTs: liver function tests, RBC: red blood cell, REMS: risk evaluation and mitigation strategy, SCr: serum creatinine, TLS: tumor lysis syndrome, VTE: venous thromboembolism, ULN: upper limit of normal, WBC: white blood cell.

**Table 3:** Commonly used first-line regimens in multiple myeloma.

	Preferred	Other Recommended	Useful in Certain Circumstances
**Transplant Eligible**	Bortezomib, lenalidomide, dexamethasone Bortezomib, cyclophosphamide, dexamethasone	Carfilzomib, lenalidomide, dexamethasone Daratumumab, lenalidomide, bortezomib, dexamethasone Ixazomib, cyclophosphamide, dexamethasone	Bortezomib, thalidomide, dexamethasone Cyclophosphamide, lenalidomide, dexamethasone Daratumumab, cyclophosphamide, bortezomib, dexamethasone Daratumumab, bortezomib, thalidomide, dexamethasone Dexamethasone, thalidomide, cisplatin, doxorubicin, cyclophosphamide, etoposide, bortezomib
**Transplant Ineligible**	***As above AND:*** Daratumumab, lenalidomide, dexamethasone Lenalidomide, dexamethasone	Carfilzomib, lenalidomide, dexamethasone Ixazomib, lenalidomide, dexamethasone Daratumumab, bortezomib, melphalan, prednisone Daratumumab, cyclophosphamide, bortezomib, dexamethasone	Bortezomib, dexamethasone Cyclophosphamide, lenalidomide, dexamethasone Carfilzomib, cyclophosphamide, dexamethasone

**Table 4:** Commonly used regimens in relapse/refractory multiple myeloma.

After 1–3 Prior Therapies	After 4 Prior Therapies
Carfilzomib, lenalidomide/pomalidomide, dexamethasone Daratumumab, bortezomib/carfilzomib, dexamethasone Daratumumab, lenalidomide/pomalidomide, dexamethasone Isatuximab-irfc, lenalidomide/pomalidomide, dexamethasone Ixazomib, lenalidomide/pomalidomide, dexamethasone Pomalidomide, bortezomib, dexamethasone Elotuzumab, bortezomib, dexamethasone Elotuzumab, lenalidomide/pomalidomide, dexamethasone	Belantamab mafodotin-blmf Selinexor, dexamethasone ± bortezomib Idecabtagene vicleucel

**Other Regimens (Less Commonly Used)**	

Venetoclax, dexamethasone [if presence of translocation (11; 14)] High dose cyclophosphamide Bendamustine, bortezomib, dexamethasone Dexamethasone, thalidomide, cisplatin, doxorubicin, Cyclophosphamide, etoposide, bortezomib	

## 4 Pharmacologic agents

### 4.1 Corticosteroids

The mechanism of action of corticosteroids is multifaceted and includes inhibition of transcription factors (NFkB, activator protein-1), upregulation of pro-apoptotic genes, downregulation of anti-apoptotic genes, and suppression of protein synthesis via inhibition of mammalian target of rapamycin (mTOR) [20]. Dexamethasone (Decadron) dosing is typically age-based with patients <75 years old receiving 40 mg intravenous (IV) or orally weekly, and patients ≥75 years old receiving 20 mg IV or orally weekly. Historically, dexamethasone doses exceeding 160 mg cumulatively in a 28-day period have been associated with an inferior progression-free survival (PFS) and overall survival (OS) due to increased toxicity (thromboembolism and infections in particular) [21]. Common adverse events with standard dosing of dexamethasone 40 mg weekly include hyperglycemia, hypertension, gastritis, insomnia, mood disturbances, and increased infections of which may be mitigated by reducing the weekly dexamethasone dose by 50%. Taking dexamethasone in the morning with breakfast will help reduce gastritis and insomnia. Dividing weekly dexamethasone dose into two doses taken on two consecutive days each week is an option for patients who are intolerant of the weekly dose. Dividing the weekly dexamethasone dose into two doses taken on two consecutive days each week is an option for patients who are intolerant of a single weekly dose.

### 4.2 Immunomodulators (IMiDs)

Currently, the three orally available immunomodulators (IMiDs) include thalidomide (Thalomid), lenalidomide (Revlimid), and pomalidomide (Pomalyst). The mechanism of action is thought to include co-stimulation of T-cells via the B7-CD28 pathway leading to increased production of interleukin 2 (IL2) and interferon gamma (IFNy), which ultimately results in downstream natural killer (NK)-cell mediated antibody-dependent cellular cytotoxicity (ADCC) towards myeloma cells [22, 23]. It is purported that IMiDs increase pro-apoptotic signaling and downregulate both osteoclastogenic signaling (IL6, tumor necrosis factor alpha [TNFa], and RANK-L).

Thalidomide is predominately metabolized via non-CYP mediated hydrolytic cleavage while pomalidomide is a substrate for CYP1A2, CYP3A4, CYP2C19, and CYP2D6. Conversely, lenalidomide has very limited hepatic metabolism and is predominately excreted renally. Hence, for patients with creatinine clearance (CrCl) < 60 mL/min, consideration needs to be taken to dose decrease or hold lenalidomide [22].

The most prevalent adverse reactions of IMiDs include myelosuppression, neuropathy, GI disturbances, teratogenicity, secondary malignancies, and venous thromboembolism (VTE). While thalidomide is typically associated with higher rates of neuropathy and VTE, lenalidomide and pomalidomide are typically more myelosuppressive [22]. Thus, the lenalidomide and pomalidomide dosing scheme incorporates a 7-day treatment free period after either 14 or 21-days of treatment depending on the regimen to allow for hematologic recovery. Thalidomide, however, is administered continuously. Providers must register patients receiving any IMiD in the Risk Evaluation and Mitigation Strategy (REMS) program through the manufacturer, which includes the following components: documentation of reproductive potential, routine negative pregnancy tests for females of childbearing age, patient awareness of teratogenicity, VTE risk, storage and handling, and inability to donate blood or sperm products [24].

### 4.3 Proteosome inhibitors (PIs)

The three commercially available proteosome inhibitors (PIs) include bortezomib (Velcade), carfilzomib (Kyprolis), and ixazomib (Ninlaro). The mechanism by which PIs induce myeloma cell death primarily includes disruption of the ubiquitin proteosome system, which disrupts regulation of downstream intracellular proteins including transcription signaling factors (i.e. nuclear factor kappa B [NF-kB]), tumor suppressor proteins (i.e. p53), and anti-apoptotic proteins (Bcl-2) [25, 26]. Bortezomib can be administered as an IV or subcutaneous (SQ) injection, however, the SQ route of administration is associated with fewer toxicities. The non-hematologic dose limiting toxicity is diarrhea, sometimes requiring dose reductions. In one phase III trial, SQ bortezomib (1.3 m/m^2^ d1, 4, 8, 11 every 21 days) produced fewer hematologic toxicities and neuropathy compared to IV bortezomib while efficacy remained similar between cohorts [27]. Bortezomib dosing is hepatically adjusted and is typically administered on a weekly basis every 28 days or on days 1, 4, 8, 11 of each 21-day cycle. In a phase II study, once-weekly bortezomib administration (1.5 mg/m^2^ on days 1, 8, 15, 22 every 28 days) was associated with fewer dose reductions and improved tolerability compared to twice-weekly bortezomib administration (1.3 mg/m^2^ on days 1, 4, 8, and 11 every 21 days) without a significant difference in efficacy [28].

Carfilzomib is administered as an IV infusion given once or twice weekly three out of four weeks of each 28-day cycle (see Table 2). In the ARROW trial, the PFS was compared between once weekly dosing and twice weekly dosing [29]. It was found that once weekly dosing had significantly prolonged PFS versus the twice weekly schedule, and safety was comparable between the two groups [29]. Once weekly dosing may be considered for more efficacy, safety, and convenience. Carfilzomib should also have a 7-day treatment-free period given the higher incidence of myelosuppression. While all three PIs cause neuropathy and cytopenias to varying degrees, carfilzomib is unique in that it is also associated with cardiopulmonary toxicity. Interstitial lung disease (1%), pulmonary hypertension (1%), and dyspnea (28%) are possible side effects [10]. While a baseline echocardiogram is not required, it may help guide the practitioner when choosing between PIs. Additionally, carfilzomib does carry a risk for tumor lysis syndrome (TLS), and hydration with 250–500 mL of IV fluid is recommended prior to each dose of the first cycle. While TLS prophylaxis with allopurinol is not routinely included, practitioners may consider monitoring for changes in uric acid and electrolytes. It should also be noted that a patient’s body surface area (BSA) should be capped at 2.2 m^2^ when dosing carfilzomib. Each PI carries a risk for peripheral neuropathy, but carfilzomib appears to be associated with less neuropathy when compared to a bortezomib-based regimen [30].

Ixazomib is the only orally available PI to date, and is typically administered once weekly on days 1, 8, and 15 of a 28-day cycle in a similar fashion to carfilzomib. Initially in the TOURMALINE–MM3 Trial, ixazomib showed a PFS benefit over placebo [31]. There is currently ongoing research and debate if ixazomib has long term benefit in the post-transplant maintenance setting.

### 4.4 Monoclonal antibodies

#### 4.4.1 Daratumumab (Darzalex)

Daratumumab is a human monoclonal antibody that targets a unique epitope on the CD38 glycoprotein and induces cell death through various Fc-dependent immune effector mechanisms. These mechanisms include complement-dependent cytotoxicity, antibody-dependent cellular cytotoxicity, antibody-dependent cellular phagocytosis and apoptosis via crosslinking [32]. Hypersensitivity reactions including rash, hives, shortness of breath, and hemodynamic changes are commonly seen with daratumumab administration, especially during the first cycle. Intravenous daratumumab carries a risk of hypersensitivity reactions of 48% whereas the risk associated with the SQ formulation is 13%. The incidence may be reduced by giving the first dose over two days, as well as adding pre-medications prior to infusion such as acetaminophen, diphenhydramine, and corticosteroids. The addition of montelukast 10 mg tablet taken daily during cycle 1 has been shown to decrease the incidence of hypersensitivity reactions with IV formulation [33]. When assessing administration techniques, SQ daratumumab route was non-inferior to IV daratumumab in terms of efficacy, pharmacokinetics, and had an improved safety profile in patients with relapsed or refractory MM [34]. Regardless of the route of administration, a corticosteroid, acetaminophen, and antihistamine should be given prior to administration. With daratumumab’s unselective ability to target CD38, this may lead to binding of CD38 on red blood cells, resulting in pan agglutination on indirect antiglobulin tests thus obscuring a patient’s blood type. To avoid significant delays in patient care, it is recommended to determine blood typing prior to first infusion [35]. If unobtainable, dithiothreitol (DTT) may be used to determine blood type post-daratumumab infusion [36].

#### 4.4.2 Elotuzumab (Empliciti)

Elotuzumab is a humanized IgG1 monoclonal antibody directed toward SLAMF7, also called CS1 (cell surface glycoprotein CD2 subset 1). SLAMF7 is highly expressed on abnormal plasma cells and natural killer cells, but not on hematopoietic stem cells. Elotuzumab directly activates natural killer cells through the SLAMF7 pathway and Fc receptors, which mediates the destruction of myeloma cells through antibody-dependent cellular cytotoxicity [37]. Elotuzumab frequency of administration is unique where it is contingent upon which IMiD is utilized (see Table 2). Safety data from the phase III ELOQUENT-2 trial assessing elotuzumab in combination with lenalidomide and dexamethasone demonstrated hypersensitivity reactions in 10% of patients in the elotuzumab arm, 70% of which occurred during the first infusion [38]. Premedication with dexamethasone (given in split dosing, see Table 2), acetaminophen, and an antihistamine within 30–60 min prior to elotuzumab are highly recommended [37].

#### 4.4.3 Isatuximab-irfc (Sarclisa)

Isatuximab-irfc induces the destruction of CD38-bearing MM cells through multiple mechanisms that include fragment crystallizable (Fc)-dependent immune effector activities supplemented by Fc-independent activities. Fc-dependent activities include NK cell-mediated antibody-dependent cellular toxicity, antibody-dependent cellular phagocytosis, and complement-dependent cytotoxicity [39]. Isatuximab-irfc also demonstrated immunomodulatory effects in vitro that may contribute indirectly to control of tumor growth in MM. In comparison with daratumumab, isatuximab-irfc binds to the CD38 epitope distinct from that targeted by daratumumab, and the possibility of isatuximab-irfc overcoming resistance to daratumumab is being explored [40]. Approach to blood typing and transfusion support while a patient is on isatuximab-irfc is identical to the recommendations with daratumumab. Isatuximab-irfc has an incremental escalation in infusion rate due to risk of hypersensitivity reactions. Patients should be given adequate hypersensitivity reaction prophylaxis constituting acetaminophen, both histamine type 1 and type 2 antagonists, and a corticosteroid. Other frequent non-hematologic adverse reactions with isatuximab-irfc include respiratory infections, cytopenias, and dyspnea.

### 4.5 Belantamab-mafodotin (Blenrep)

Belantamab is a novel anti-BCMA antibody conjugated to cytotoxic monomethyl auristatin. This mechanism allows the delivery of the cytotoxic agent to selected tumor cells while eliciting a host immune response simultaneously [41]. It is the first-in-class biologic for patients who have previously attempted four other treatments, including an anti-CD38 monoclonal antibody, a proteosome inhibitor, and an immunomodulatory agent. Belantamab has historically been administered to relapsed/refractory MM patients in combination with dexamethasone alone, however, a recent study evaluated belantamab in combination with bortezomib and dexamethasone. Overall response rate (ORR) was 78% with very good partial response of 50% and partial response in 28% of patients [42]. In terms of safety, Belantamab is associated with a high incidence of keratopathy. To mitigate such risks, an ophthalmic exam is required through its REMS program prior to and during therapy to assess baseline vision and possible adverse eye effects. Preservative-free lubricating eye drops at least four times daily are prescribed during therapy to prevent dryness.

### 4.6 Selinexor (Xpovio)

Selinexor encompasses a novel mechanism of action which includes reversible inhibition of the nuclear exportin 1 (XPO1) transporter protein which is overexpressed in MM [43]. Endogenously, XPO1 shuttles tumor suppressor proteins including but not limited to, p53, retinoblastoma, nucleophosmin, and p73 from the nucleus to the cytoplasm. Ultimately, disruption of XPO1 function leads to accumulation of tumor suppressor proteins within the nucleus, which slows oncogene transcription. Initially, twice-weekly selinexor (see Table 2) was FDA-approved in combination with dexamethasone in adults with triple-class refractory MM [43]. Most recently, the combination of weekly selinexor (see Table 2), bortezomib, and dexamethasone was FDA-approved in December 2020 for the treatment of adults with MM who have received at least one prior line of therapy. Adverse reactions most commonly include nausea/vomiting, thrombocytopenia, anemia, hyponatremia, and hypophosphatemia [18]. In the STORM trial, nausea rates (any grade) were reported to be 72% despite premedication with ondansetron or equivalent before each selinexor dose [44]. The addition of other antiemetics such as olanzapine or neurokinin-1 receptor antagonists may be considered in clinical practice. Incorporating olanzapine (5–10 mg daily taken on the evening prior to selinexor dosing day and for three days thereafter) with 5HT3-recetor antagonist premedication may reduce nausea and vomiting rates.

### 4.7 Venetoclax (Venclexta)

Venetoclax is an orally bioavailable BCL-2 inhibitor that induces cell death in MM cells. In the BELLINI study, venetoclax 800 mg daily was given in combination with bortezomib and dexamethasone to PI treatment naïve relapsed/refractory (R/R) MM patients [45]. The addition of venetoclax displayed improvement in relative response (RR) and PFS, particularly in patients harboring translocation (11;14) or BCL2^high^ gene expression. Of note, patients with high-risk cytogenetics and BCL2^high^ expression in the absence of translocation (11;14) were most at risk when treated with venetoclax and favored the placebo arm [45]. While current clinical trials are investigating the utility of venetoclax in combination with PIs and anti-CD38 targeting antibodies, venetoclax currently remains off-label for the indication of MM. Serious adverse reactions associated with venetoclax use include neutropenia, thrombocytopenia, anemia, diarrhea, and pneumonia. Best clinical judgment is needed in determining TLS prophylaxis and monitoring. Venetoclax also carries several drug-drug interactions and a thorough medication review should be performed prior to initiation.

## 5 Cellular therapies

### 5.1 Autologous stem cell transplant

Autologous stem cell transplant (auto-HSCT) has become a fundamental treatment in the management of newly diagnosed MM (NDMM), particularly in patients who are young and fit. While allogeneic stem cell transplant may play a role in MM, it is typically reserved for patients with relapse/refractory disease. Auto-HSCT has yielded high rates of efficacy, with a complete remission in one-third of transplant recipients, and a median PFS of 18–27 months without further therapy [46]. With the addition of novel agents supporting a deepened and sustained response in patients, the optimal timing of auto-HSCT has become a topic of debate. The International Myeloma Working Group recommends that auto-HSCT should be offered at some point in the course of treatment program for a patient eligible to receive high dose chemotherapy prior to auto-HSCT [47]. Advantages of early auto-HSCT confer benefits related to decreased exposure to chemotherapy toxicities and lesser financial burden with novel therapy use, however achieving minimal residual disease negativity prior to auto-HSCT has become more of a priority recently. In a study conducted by Munshi and colleagues, the median PFS was 61 months for patients proceeding to auto-HSCT with minimal residual disease negativity compared to 24.1 months for patients who were minimal residual disease positive [48]. Age and performance status should be considered if patients opt to defer auto-HSCT for the relapse setting. As such, it is safe to conclude that auto-HSCT can improve outcomes in the first line or relapsed/refractory setting, and frontline auto-HSCT remains the standard of treatment for fit, young, and select elderly patients with newly diagnosed multiple myeloma.

Maintenance therapy has displayed success in delaying disease relapse and prolonging survival in the post-transplant setting. IMiDs and PIs have been utilized in the maintenance setting, with lenalidomide being the preferred option. Multiple phase 3 clinical trials have demonstrated the benefits of lenalidomide maintenance therapy in patients with NDMM for outcomes including PFS and OS [49]. Bortezomib has also displayed efficacy in the high-risk population, particularly in patients with chromosome 17p13 deletion and those with kidney disease [50]. As previously mentioned, provider discretion should be used, and patients should be informed of survival data when using ixazomib in the post-transplant maintenance setting. The ideal duration of maintenance therapy remains unknown, and the current recommendation is to continue therapy until there is evidence of disease progression, especially in patients with high-risk disease [50]. In patients with high-risk MM, post-transplant consolidation with the combination of an IMiD, PI, corticosteroid, and/or an anti-CD38 targeting monoclonal antibody may become more commonly utilized [50].

### 5.2 Chimeric antigen receptor (CAR) T-Cell therapy

In March 2021, the FDA-approved the first commercial CAR T-cell therapy, idecabtagene vicleucel (ABECMA), for the indication of relapsed/refractory MM after four or more lines of therapy including an IMiD, PI, and anti-CD38 monoclonal antibody [51]. Idecabtagene vicleucel consists of a CAR T-cell construct targeting B-cell maturation antigen (BCMA) which is expressed on the surface of both normal and malignant plasma cells, promoting survival of myeloma cells. This approval was based off the results from the phase II KarMMa study by Mushni NC and colleagues which demonstrated an ORR of 73% [52]. More recently, a second BCMA-targeting CAR T-cell therapy, ciltacabtagene autoleucel (CARVYKTI), was FDA-approved for relapsed/refractory MM after four or more lines of therapy [53]. This was based off the results from the phase II CARTITUDE-2 trial of which reported an ORR of 95% [54].

Like other CAR T-cell products, cytokine release syndrome (CRS) and neurotoxicity were the most prevalent toxicities with both idecabtagene vicleucel and ciltacabtagene autoleucel [51, 53]. Due to the potential for neurologic toxicity (decreased consciousness, altered mental status, seizures), patients should receive anti-seizure prophylaxis (i.e. levetiracetam) per institutional standard for at least 8 weeks following CAR T-cell infusion [51]. Providers should also be monitoring for pseudo-parkinsonism, a unique neurotoxicity symptom of anti-BCMA CAR T-cell therapy. As infections were prevalent in the anti-BCMA CAR T-cell trials, providers should ensure patients receive herpes simplex/varicella virus (HSV/VZV) prophylaxis and pneumocystis jiroveci (PJP) prophylaxis during and after CAR T-cell infusion. Patients should receive antifungal prophylaxis in alignment with NCCN recommendations [55]. Facility-specific enrollment in a REMS program is required prior to utilization of both products to ensure providers are competent in recognition and management of CRS and neurotoxicity.

## 6 Supportive care

### 6.1 Infection prevention

According to the National Comprehensive Cancer Center (NCCN) guidelines for MM, patients who are receiving therapy with PIs, daratumumab, isatuximab-irfc, elotuzumab, or high dose dexamethasone (doses exceeding 160 mg cumulatively per cycle) should receive HSV/VZV prophylaxis with either acyclovir or valacyclovir [3]. NCCN also recommends both PJP and fungal prophylaxis for patients receiving high dose dexamethasone. PJP prophylaxis with sulfamethoxazole/trimethoprim is the preferred strategy, however dapsone, atovaquone, and pentamidine are alternative agents for patients with clinically significant drug-drug interactions, hypersensitivity, or intolerance with sulfamethoxazole/trimethoprim [56]. Providers may also consider three months of fluoroquinolone prophylaxis during induction therapy for patients with a high risk of infection as this has been associated with a reduction in febrile episodes and death compared to placebo [57]. Recently the American Society of Clinical Oncology (ASCO) endorsed that all patients anticipating systemic therapy for cancer should be tested for HBV at baseline [58]. Patients found to have chronic HBV at baseline should receive antiviral prophylaxis with either entecavir, tenofovir disoproxil fumarate, or tenofovir alafenamide fumarate throughout systemic anticancer therapy and for a duration of 12 months after. Finally, providers may consider administration of IV immunoglobulin therapy in MM patients with recurrent serious infections in the setting of hypogammaglobinemia (IgG <400 mg/dL) [3].

### 6.2 Bone health

Pathologic fractures are a significant cause of morbidity in patients with MM. In one population-based study, the incidence of pathologic fractures was 8.7% at MM diagnosis and 23.1% after diagnosis [59]. NCCN guidelines currently endorse use of denosumab (Xgeva) therapy or use of an IV bisphosphonate such as zoledronic acid (Zometa) for up to two years from diagnosis, even in the absence of osteolytic lesions [3]. Of note, monthly denosumab therapy has been shown to be non-inferior to monthly zoledronic acid therapy in delaying time to first skeletal-related event in newly diagnosed patients with MM [60].

Although no significant difference in the incidence of osteonecrosis of the jaw (ONJ) was detected between cohorts, zoledronic acid was associated with higher rates of renal toxicity [60]. Selection of a bone-modifying therapy should consider route of administration, frequency, safety, and expense. Both agents should be avoided in patients with a history of osteonecrosis, and zoledronic acid use should be avoided in patients with significant renal insufficiency. Zoledronic acid is given IV and is renally adjusted down to a CrCl of 30 mL/min [61]. In comparison, denosumab is a subcutaneous injection that is administered as a flat dose of 120 mg regardless of renal function, but the expense of denosumab exceeds that of zoledronic acid which may preclude denosumab use [62]. Additionally, zoledronic acid may be preferred given the option for reduced interval dosing given its long duration of action. No significant difference in the incidence of skeletal-related events was seen when comparing zoledronic acid given monthly vs. every 3 months in patients with MM, metastatic breast cancer, or metastatic prostate cancer [63].

Continuation of bone-modifying therapy beyond two years should be a patient-specific decision. DEXA and bone scans obtained two years after start of bone-modifying therapy is useful when determining subsequent frequency of therapy. Evidence of osteolytic lesions would warrant continuation of bisphosphonate therapy every one to three months or monthly denosumab. If a patient has no evidence of osteolytic lesions but is noted to have osteopenia or osteoporosis, consideration could be taken to de-escalate bone-modifying therapy to the FDA-approved frequency of administration for those indications. Prior to starting bone-modifying therapy, providers should ensure patients are taking a minimum of 500 mg per day of elemental calcium and 400 IU per day of vitamin D provided there is no evidence of hypercalcemia. A baseline dental examination is recommended prior to starting bone-modifying therapy as bone modifying therapy should be held 8–12 weeks before procedures and resumed only once healing is complete to reduce the risk of ONJ [64, 65]. Bone-modifying therapy should be held for a similar duration around other major bone surgeries as well. Patients should also be encouraged to maintain adequate oral hygiene as this has been shown to reduce the risk of ONJ [66].

### 6.3 Thromboprophylaxis

MM patients have the highest risk for VTE in the first 6 months following new diagnosis of MM [3]. The thrombogenicity of myeloma is multifactorial, with disease- and treatment-related factors playing important roles. Immunomodulatory drugs and high dose dexamethasone are known to enhance the thrombotic potential [67]. The IMPEDE or SAVED scoring tools endorsed by NCCN can help providers identify which patients qualify for thromboprophylaxis based on underlying risk factors and the current MM therapy the patient is receiving. Patients with a low-risk score for VTE are candidates for aspirin 81–325 mg daily while patients with a high-risk score for VTE are candidates to receive low-molecular weight heparin (LMWH), a direct oral anticoagulant therapy (DOAC), or warfarin assuming no major contraindications. A patient should remain on thromboprophylaxis if the risk factors for VTE remain present.

## 7 Discussion

Treatment options for MM continue to evolve as new therapeutic strategies with novel mechanisms arise. Navigating this landscape as a new advanced practice provider can be challenging as the ideal sequence of MM therapy is not well elucidated. Recently, the general approach to induction therapy incorporated the use of at triple regimen consisting of corticosteroids, PIs, and iMiDs. However, new data suggests the addition of anit-CD38 targeting monoclonal antibodies to induction leads to increased minimal residual disease (MRD)-negativity rates in transplant-eligible patients [68, 69]. Considering this, quadruple therapy induction regimens may become the preferred. Auto-HSCT followed by consolidation and maintenance therapy remains an essential component of standard of care MM treatment. As evidenced previously, data by Munshi and colleagues relays the impact of consolidative auto-HSCT on long-term survival outcomes in MM [52]. Choices for subsequent-line should be based on tolerability, feasibility, cost, and side effect profile, and CAR-T therapy remains a viable option for quadruple-refractory patients. This review aimed to familiarize new advanced practice providers with the intricacies of each therapeutic drug class as well as provide a general overview of the role of cellular therapy in this setting. Advanced practice providers also play a key role in implementing supportive care strategies in this population such as infection prevention, thromboprophylaxis, and bone health.

Naturally, there remains several unmet needs within this landscape of MM. First, the use of advanced minimal residual disease (MRD) testing using next-generation sequencing (NGS) technology with a minimum sensitivity of 1 in 10^5^ nucleated cells is becoming more common place given studies suggesting this technology serves as an important prognostic factor [70]. While MRD testing via NGS is most commonly performed both at the end of induction therapy and post-auto-HSCT, the optimal timing of MRD testing in treating patients with relapsed MM remains a question [71]. Second, the sequencing of therapies in the relapsed MM patient population who have received an anti-BCMA targeting therapy (CAR-T therapies, belantamab-mafodotin) remains a debate. What is the optimal duration between receiving BCMA-targeting CAR-T therapy and transitioning to other BCMA-targeting therapies such as teclistamab, a newer monoclonal antibody currently under investigation [72]? Will BCMA expression testing become more commonplace to help guide practitioners with these decisions? A new practitioner may need to consider such questions in near future.

## 8 Conclusion

The management of patients with MM remains a challenge given the complexities in therapy and the significant morbidity associated with this malignancy. Understanding the pathophysiology of MM and the pharmacology of the therapies used is crucial to providing excellent care. This review aims to serve as a succinct tertiary reference for new advanced practice providers.

## Declarations

### Author contribution statement

All authors listed have significantly contributed to the development and the writing of this article.

### Funding statement

This research did not receive any specific grant from funding agencies in the public, commercial, or not-for-profit sectors.

### Data availability statement

No data was used for the research described in the article.

### Declaration of interests statement

The authors declare no conflict of interest.

### Additional information

No additional information is available for this paper.
